# Empyema Caused by Pasteurella multocida in a Patient With Chronic Obstructive Respiratory Disease Taking Inhaled Corticosteroids: A Case Report

**DOI:** 10.7759/cureus.35156

**Published:** 2023-02-18

**Authors:** Hidesato Odaka, Ruriko Asahi, Kengo Shimada, Motonari Kamei, Taisei Kato

**Affiliations:** 1 Department of Respiratory Medicine, Japanese Red Cross Akita Hospital, Akita, JPN; 2 Post Graduate Clinical Education Center, Japanese Red Cross Akita Hospital, Akita, JPN; 3 Department of Bacteriological Examination, Japanese Red Cross Akita Hospital, Akita, JPN

**Keywords:** pet, cat, inhaled corticosteroids, chronic obstructive respiratory disease, empyema, pasteurella multocida

## Abstract

*Pasteurella multocida* (*P. multocida*) infection develops in patients with chronic obstructive pulmonary disease (COPD). Inhaled corticosteroids (ICS) are used for the treatment of COPD. Herein, we report a case of empyema caused by *P. multocida* in a patient using ICS for COPD. A 79-year-old man with COPD presented with general fatigue. He was treated with triple therapy including ICS. Contrast-enhanced computed tomography revealed encapsulated pleural effusion in the left chest. We initiated antibiotics, sulbactam sodium/ampicillin sodium (3 g × 4), and thoracic drainage. His pleural effusion culture turned out positive and *P. multocida *was detected. The patient was diagnosed with empyema caused by *P. multocida*. The triple therapy combination, including ICS, was changed to a double therapy combination without ICS. The subsequent progress was relatively good, and on the 49th day of hospitalization, the patient was discharged. The onset of *P. multocida* infection may be associated with ICS use, which may best be avoided in a patient with COPD who is at risk of *P. multocida* infection.

## Introduction

*Pasteurella multocida* (*P. multocida*) is a gram-negative coccobacillus and zoonotic microbe found in the oral flora of cats (70%), dogs, and other mammals [1,2]. Most patients with *P. multocida* infection have an underlying cardiopulmonary disease and/or are immunocompromised (57%) [3]. Chronic obstructive pulmonary disease (COPD) is an underlying cardiopulmonary disease, and inhaled corticosteroids (ICS) are sometimes used for its treatment; however, ICS can increase the risk of pneumonia [4]. The association between *P. multocida* infection and ICS use in patients with COPD is ambiguous. Herein, we report a case of empyema caused by *P. multocida* in a patient using ICS for COPD.

## Case presentation

A 79-year-old man with COPD (Global Initiative for Chronic Obstructive Lung Disease (GOLD) stage 3) presented with general fatigue. He had smoked 20 cigarettes per day for 52 years (from 25 to 77 years of age) and was treated with a triple therapy combination of a long-acting muscarinic antagonist (LAMA; umeclidinium: 62.5 µg), a long-acting beta2-agonist (LABA; vilanterol: 25 µg), and ICS (fluticasone furoate: 100 µg) for over six months. He had experienced one episode of COPD exacerbation in the preceding year. However, he had no history of tuberculosis and no history or complications of asthma. He had received a kitten from an acquaintance one month before onset. However, he did not allow the kitten to bite him.

On admission, his vital parameters were as follows: blood pressure of 108/72 mmHg, pulse rate of 96 beats/minute, respiratory rate of 20 breaths/minute, O_2_ saturation of 89% on room air, and temperature of 37.6°C. No pulmonary murmurs, abdominal vascular murmurs, or other remarkable physical findings were noted. Abnormal laboratory findings on admission were as follows: white blood cell count of 22,400/µL, C-reactive protein level of 28.72 mg/dL, and procalcitonin level of 4.01 ng/mL (Table 1).

**Table 1:** Laboratory findings at the first hospital visit Alb, albumin; ALT, alanine transaminase; AST, aspartate aminotransferase; BUN, blood urea nitrogen; Cl, chloride; Cr, creatinine; CRP, C-reactive protein; HbA1c, hemoglobin A1c; HGB, hemoglobin; HCT, hematocrit; K, potassium; LDH, lactate dehydrogenase; Na, sodium; PCR, polymerase chain reaction; PCT, procalcitonin; PLT, platelet; RBC, red blood cell; T-SPOT, tuberculosis screening test; TP, total protein; WBC, white blood cell.

Laboratory investigation	Results	Reference range
WBC count	22,400/μL	3,300–8,600/μL
Neutrophils	89.0%	41.2–69.7%
Lymphocytes	5.0%	22.1–46.9%
Monocytes	6.0%	4.1–9.6%
Eosinophils	0.0%	0.0–3.5%
Basophils	0.0%	0.0–1.1%
RBC count	298 × 10^4^/μL	386–492 × 10^4^/μL
HGB	8.2 g/dL	11.6–14.8 g/dL
HCT	24.0%	35.1–44.4%
PLT	47.7 × 10^4^/μL	15.8–34.8 × 10^4^/μL
TP	6.4 g/dL	6.6–8.1 g/dL
Alb	2.0 g/dL	4.1–5.1 g/dL
AST	23 IU/L	13–30 IU/L
ALT	16 IU/L	7–23 IU/L
LDH	153 IU/L	124–222 IU/L
BUN	38.9 mg/dL	8.0–20.0 mg/dL
Cr	1.59 mg/dL	0.46–0.79 mg/dL
Na	134 mEq/L	138–145 mEq/L
K	4.2 mEq/L	3.6–4.8 mEq/L
Cl	98 mEq/L	101–108 mEq/L
CRP	28.72 mg/dL	0.00–0.14 mg/dL
PCT	4.01 ng/mL	≤0.05 ng/mL
T-SPOT	(-)	(-)
HbA1c	6.2%	4.6–6.2%
Acid-fast test sputum smear	(-)	(-)
Acid-fast test sputum culture	(-)	(-)
Acid-fast test PCR	(-)	(-)
Sputum culture	*Pseudomonas aeruginosa* 1+	(-)
Blood culture	(-)	(-)

**Figure 1:**
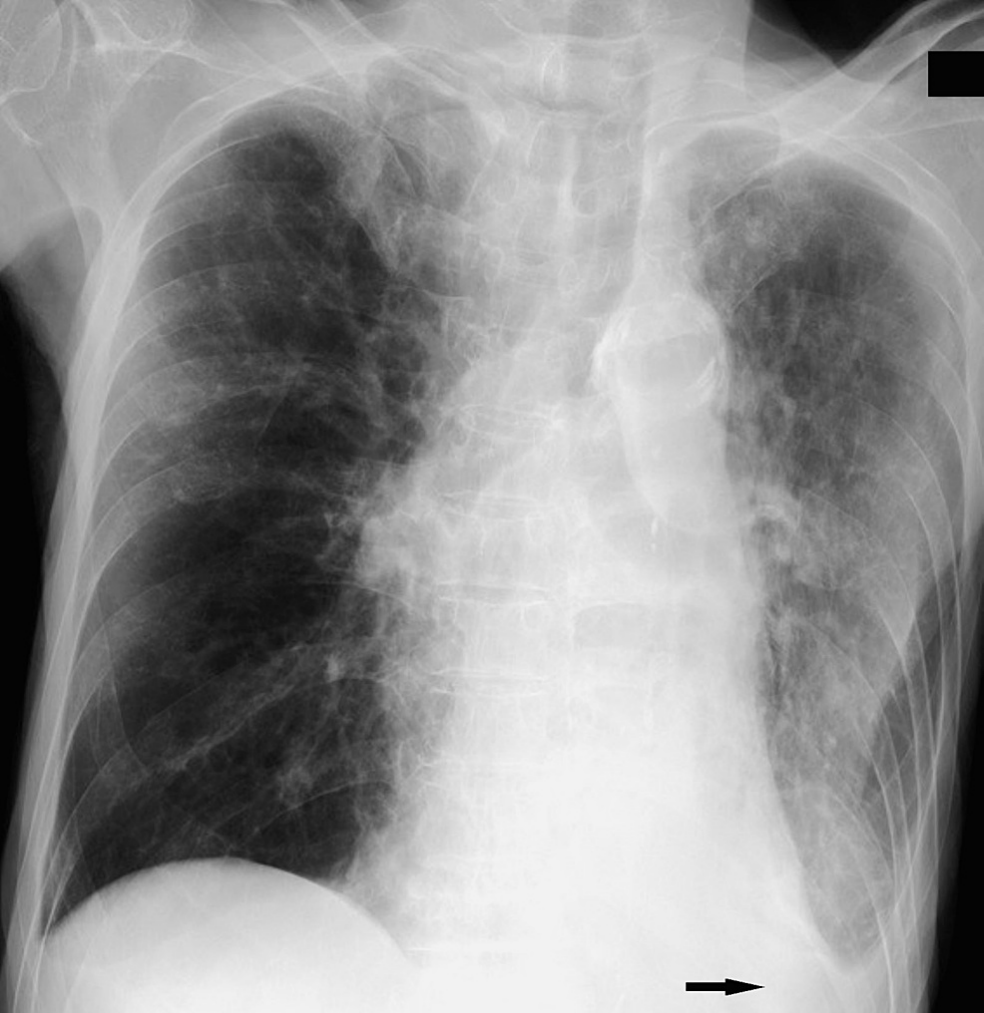
Chest radiograph on admission A chest radiograph on admission showed increased permeability of the right lower lung fields, widespread decreased permeability of the left lung fields, and left pleural effusion (arrow).

A plain chest radiograph revealed decreased permeability in the left lung fields, and the costophrenic angle was dull, indicating pleural effusion (Figure 1).

**Figure 2:**
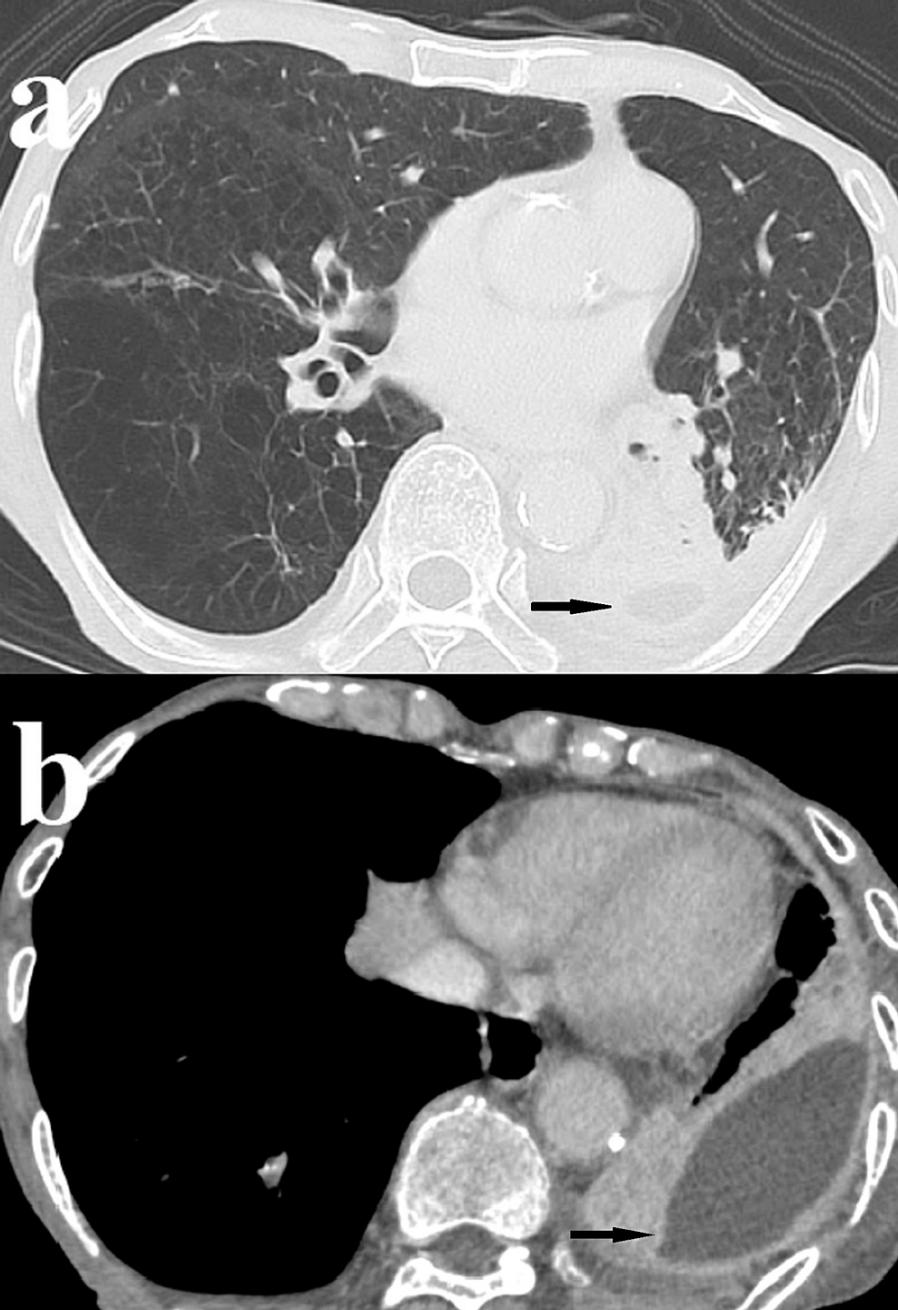
Contrast-enhanced computed tomography scan Contrast-enhanced CT scan showing (a) pulmonary emphysema of the right lung lower lobe and pleural effusion in the left dorsal chest (arrow), and (b) encapsulated pleural effusion in the left lateral chest (arrow).

Contrast-enhanced computed tomography revealed encapsulated pleural effusion in the left dorsal and lateral chest (Figure 2).

We performed thoracentesis of the left lateral chest, and the pleural effusion was purulent (Table 2).

**Table 2:** Laboratory findings of the pleural effusion fluid ADA, adenosine deaminase; Ex, exudative; LDH, lactate dehydrogenase; T-cho, total cholesterol; Trans, transudative.

Laboratory investigation	Results	Reference range
Color	Purulent	Not available
Cell count	450,549/µL	<250/µL
Polymorphonuclear	81%	Not available
Lymphocytes	15%	Not available
Other	4%	Not available
Histiocytes	(+)	Not available
Specific gravity	1.026	Not available
Rivalta	(-)	Ex (+) Trans (-)
Protein	3.3 g/dL	Ex >3 g/dL
Glucose	0 mg/dL	>60 mg/dL
pH	6.743	>7.3
LDH	39,921 mg/dL	Not available
T-cho	30 mg/dL	Not available
ADA	Unable to inspect	<37 mg/dL
Cytology	Negative	Negative
Mycobacterium tuberculosis	Negative	Negative
Bacteria	Pasteurella multocida	Negative

**Figure 3:**
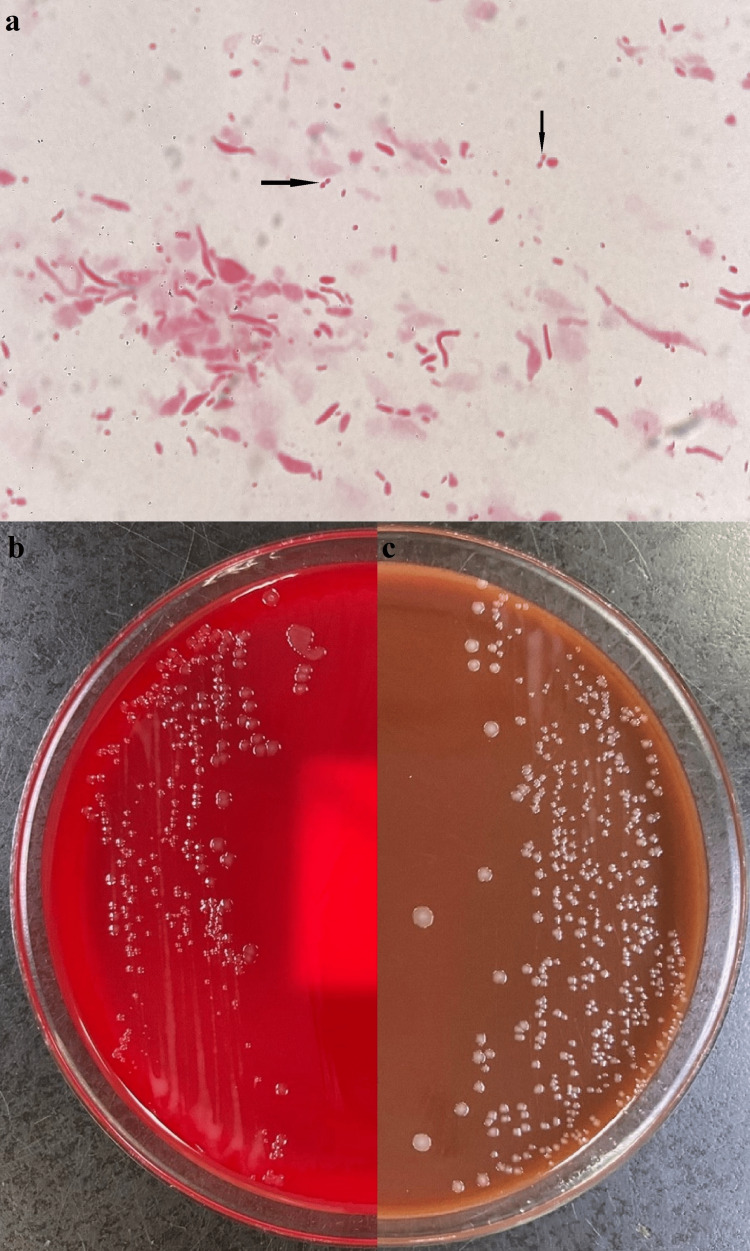
Gram smear and agar plate Gram smear from the growth, culture on a blood agar plate, and culture on chocolate agar plate showing (a) gram smear from the growth showing gram-negative coccobacilli (arrow); (b) blood agar plate (sheep) showing smooth grey colonies of *Pasteurella multocida*; and (c) chocolate agar plate showing smooth grey colonies of *P. multocida.*

We initiated the patient on antibiotic treatment with sulbactam sodium/ampicillin sodium (3 g × 4), and thoracic drainage was performed from the left lateral chest. Pleural effusion culture was positive and yielded *P. multocida*; thus, the patient was diagnosed with empyema caused by *P. multocida* (Figure 3).

We judged the *Pseudomonas aeruginosa* cultured from the sputum to have colonized the respiratory tract and changed sulbactam sodium/ampicillin sodium (3 g × 4) to benzylpenicillin potassium (2 million units × 6) based on antimicrobial susceptibility testing. We also performed thoracic drainage from the left dorsal chest. The subsequent progress was relatively good, and on the 49th day of hospitalization, the patient was discharged. The triple therapy combination of LAMA-LABA-ICS for GOLD 3 COPD was changed to a double therapy combination of LAMA-LABA after admission; however, no acute exacerbation of COPD occurred during hospitalization.

## Discussion

The course of this patient suggested two important clinical issues. First, the onset of *P. multocida* infection may be associated with ICS use. Second, the use of ICS may be best avoided in a patient with COPD who is at risk of *P. multocida* infection.

First, the onset of *P. multocida* infection may be associated with ICS use. A systemic review and meta-analysis of 11 randomized controlled trials on patients with stable COPD undergoing a ≥ six-month-long ICS therapy revealed that ICS therapy was associated with a 34% increased risk of pneumonia, although the causative bacteria have not been described in detail. A potential explanation of the mechanism is that ICS therapy may increase the pneumonia risk in patients with COPD by increasing local airway immunosuppression [4,5]. Moreover, compared with monotherapy, combined therapy with an ICS and a LABA may increase the distal ICS delivery to the alveolar bed, which can further propagate the immunosuppressive effects of ICS [4,6,7]. In our case, the patient had no history of a pet bite; therefore, it was presumed that the patient's *P. multocida* infection was endogenous or secondary to contact with secretions of their pet [8], although the most common infections caused by *P. multocida* are local wound infections following animal bites or scratches [2]. The onset of *P. multocida* infection was attributed not only to COPD but also to the use of an ICS and a LABA.

In our case, the ICS used was fluticasone furoate at 100 µg per day. However, even low doses of fluticasone (<500 µg per day) in patients with COPD are associated with an increase in the risk of severe pneumonia, if they are current users of ICS [9]. Conversely, the risk of severe pneumonia with budesonide is relatively lower even at high doses. This is because compared with budesonide, fluticasone is more potent (i.e., has a greater effect on intracellular steroid receptors), more lipophilic, and has a longer half-life [9,10].

Additionally, regarding COPD medications, we changed LAMA-LABA-ICS to LAMA-LABA because the risk of pneumonia was significantly higher with triple therapy than with umeclidinium-vilanterol in the IMPACT clinical trial [11].

Second, the use of ICS may be best avoided in a patient with COPD who is at risk of *P. multocida* infection. Agusti et al. listed several factors to consider when initiating ICS treatment in patients with COPD [12]. These factors include strong support, consider-use, and avoid-use. Strong support includes a history of hospitalization for exacerbation of COPD, two or more moderate exacerbations of COPD per year, blood eosinophil count >300 cells/µL, and a history of concomitant asthma. The indications for ICS treatment include one moderate exacerbation of COPD per year and a blood eosinophil count of 100-300 cells/µL. Avoid use includes repeated pneumonia events, blood eosinophil count <100 cells/µL, and history of mycobacterial infection. By this classification, our patient was categorized into the consider-use group before admission. However, considering the risk of *P. multocida* infection, the use of ICS might have been avoided owing to the high mortality associated with *P. multocida* empyema (45%) [3]. In a patient with COPD, especially one who has a cat or a dog, ICS use can lead to the onset of a *P. multocida* infection. The risk/benefit ratio of adding ICS should be carefully considered in each patient [12].

One may opine that patients with COPD should give up their pets. According to the Centers for Disease Control and Prevention guidelines, there is no need to part with most pets; however, it is important to understand the risks involved and avoid unnecessary exposure [13,14].

## Conclusions

ICS use may contribute to the onset of *P. multocida* infections and may be best avoided in patients with COPD who are at risk of these infections. We must be cautious whether patients using ICS for COPD have a history of unnecessary exposure to cats or dogs. Further reports should be accumulated to determine whether the risk of using ICS in patients with COPD may be present more frequently.
